# Dapagliflozin once‐daily and exenatide once‐weekly dual therapy: A 24‐week randomized, placebo‐controlled, phase II study examining effects on body weight and prediabetes in obese adults without diabetes

**DOI:** 10.1111/dom.12779

**Published:** 2016-09-26

**Authors:** Per Lundkvist, C. David Sjöström, Sam Amini, Maria J. Pereira, Eva Johnsson, Jan W. Eriksson

**Affiliations:** ^1^ Department of Medical SciencesUppsala UniversityUppsalaSweden; ^2^ AstraZenecaGothenburgSweden

**Keywords:** dapagliflozin, exenatide, obesity, prediabetes

## Abstract

**Aims:**

To explore the effects of dual therapy with dapagliflozin and exenatide on body weight, body composition, glycaemic variables and systolic blood pressure (SBP) in obese adults without diabetes.

**Materials and methods:**

In this single‐centre, double‐blind trial, we randomized 50 obese adults without diabetes (aged 18–70 years; body mass index 30–45 kg/m^2^) to oral dapagliflozin 10 mg once daily plus subcutaneous long‐acting exenatide 2 mg once weekly or placebo. MRI was used to assess change in body composition. Participants were instructed to follow a balanced diet and exercise moderately.

**Results:**

Of 25 dapagliflozin/exenatide‐ and 25 placebo‐treated participants, 23 (92.0%) and 20 (80.0%) completed 24 weeks of treatment, respectively. At baseline, the mean participant age was 52 years, 61% were female, the mean body weight was 104.6 kg, and 73.5% of participants had prediabetes (impaired fasting glucose or impaired glucose tolerance). After 24 weeks, for dapagliflozin/exenatide versus placebo: the difference in body weight change was −4.13 kg (95% confidence interval −6.44, −1.81; *P* < .001), which was mostly attributable to adipose tissue reduction without lean tissue change; 36.0% versus 4.2% of participants achieved ≥5% body weight loss, respectively; and prediabetes was less frequent with active treatment (34.8% vs 85.0%, respectively; *P* < .01). The difference in SBP change for dapagliflozin/exenatide versus placebo was −6.7 mm Hg. As expected, nausea and injection‐site reactions were more frequent with dapagliflozin/exenatide than with placebo. Only two and three participants, respectively, discontinued because of adverse events.

**Conclusions:**

Compared with placebo, dapagliflozin/exenatide dual therapy reduced body weight, frequency of prediabetes and SBP over 24 weeks and was well tolerated in obese adults without diabetes.

## 1 INTRODUCTION

Globally, more than 1.9 billion adults were overweight [body mass index (BMI) ≥25 kg/m^2^] in 2014, and of these, 600 million were obese (BMI ≥30 kg/m^2^).^1^ The management of obesity is challenging; dietary and lifestyle interventions are difficult to sustain,^2^, ^3^ and pharmacotherapies for obesity have generally been ineffective over time, often with troublesome side effects.^4^

In contrast to older glucose‐lowering medications (sulphonylureas, insulin, thiazolidinediones) that cause body weight gain,^5^ newer treatments for type 2 diabetes (T2D) are associated with body weight loss, including selective sodium‐glucose co‐transporter‐2 (SGLT2) inhibitors (e.g. dapagliflozin, empagliflozin) and glucagon‐like peptide‐1 receptor agonists (GLP‐1RAs; e.g. exenatide, liraglutide). Their glucose‐lowering activities are blood glucose‐dependent, which minimizes hypoglycaemia, but they produce sustained body weight loss via different mechanisms.^6^, ^7^ SGLT2 inhibitors reduce renal glucose reabsorption, thereby inducing glucosuria. The resulting excretion of calories and mild diuresis are associated with body weight loss and systolic blood pressure (SBP) reduction.^8^, ^9^, ^10^ GLP‐1RAs reduce body weight by decreasing food intake as a result of centrally mediated reduced appetite, with slowed gastric emptying also contributing.^7^ SBP reductions also occur with GLP‐1RAs.^11^

Body weight loss with dapagliflozin, a first‐in‐class highly selective SGLT2 inhibitor, is consistently observed among patients with T2D receiving oral dapagliflozin 10 mg once daily as monotherapy^12^ or with additional glucose‐lowering therapies.^13^, ^14^, ^15^, ^16^, ^17^, ^18^ Mean body weight loss versus placebo, estimated from a network meta‐analysis, is −2.2 kg over 24 weeks,^19^ mostly accounted for by decreased body fat mass.^14^ Clinical data over 2 years show that body weight reduction with dapagliflozin 10 mg once daily is maintained.^20^

Exenatide, the first‐in‐class GLP‐1RA, is available in a subcutaneously injected extended‐release (2 mg once weekly) formulation. In patients with T2D, mean body weight reduction with exenatide 2 mg once weekly versus placebo, estimated from a mixed treatment comparison meta‐analysis, is about −1.6 kg over 24 weeks,^21^ mostly as a result of decreased body fat mass.^22^ Data from 3 and 5 years of treatment show sustained body weight loss with exenatide 2 mg once weekly.^23^, ^24^

Combination therapy with agents acting via different mechanisms may be the most effective pharmacological approach to treating obesity and addressing evolutionary counter‐regulatory mechanisms that maintain body weight.^25^ For example, calorie loss through increased urinary glucose excretion with SGLT2 inhibitor treatment may lead to increased appetite,^8^ which might be countered by a GLP‐1RA. Combining these treatments seems appropriate to enhance and sustain body weight loss.

The present proof‐of‐concept study examined body weight loss during dual therapy with oral dapagliflozin 10 mg once daily and subcutaneous exenatide 2 mg once weekly in obese participants without diabetes. Body composition, glycaemic control, and blood pressure were also examined. To the best of our knowledge, this is the first report of a randomized controlled trial evaluating initiation of dual therapy with an SGLT2 inhibitor and a GLP1‐RA.

## 2 MATERIALS AND METHODS

### 2.1 Study design and participants

**Figure 1:**
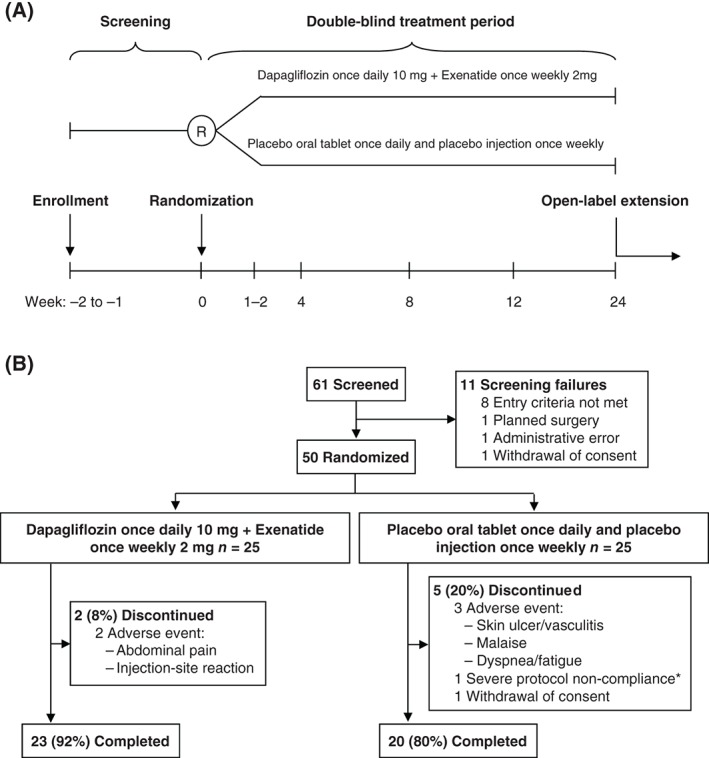
A, Study design and B, CONSORT flow diagram. *A profound lifestyle change, including a strict low‐carbohydrate/high‐fat diet that elevated blood ketones, and resulted in exclusion of this patient from the full analysis set.

In this collaborative, investigator‐sponsored, 24‐week, single‐centre, randomized, parallel‐group, double‐blind, placebo‐controlled phase IIa study (Figure 1A), we evaluated the efficacy and safety of dual therapy with dapagliflozin 10 mg once daily and exenatide 2 mg once weekly in obese participants without diabetes (ClinicalTrials.gov identifier: NCT02313220). The study was conducted at a single centre in Sweden from December 2014 until August 2015. An optional 28‐week, open‐label extension followed, to be reported separately. Obese men and women aged 18 to 70 years, without diabetes and with a BMI of 30 to 45 kg/m^2^, were eligible for enrolment (for full inclusion/exclusion criteria, see File S1).

All participants provided written informed consent before enrolment. The protocol was approved by the Independent Regional Ethics Committee in Uppsala, Sweden, and by the Swedish Medical Products Agency. The study was conducted in accordance with the Declaration of Helsinki and the International Conference on Harmonisation/Good Clinical Practice guidelines.

Participants were enrolled at a screening visit 1 to 2 weeks before randomization at week 0. Thereafter, follow‐up visits occurred at weeks 4, 8, 12 and 24.

### 2.2 Randomization and blinding

Eligible participants were randomized 1 : 1 to receive active treatment or matching placebo using computer codes independently generated by PCG Clinical Services AB (Uppsala, Sweden) and sequentially assigned by the study investigator. Product identity was concealed using sequentially numbered containers. Random assignment to study treatment was stratified by sex and predefined to generate a study population comprising 60% women and 40% men so as to avoid the >80% female enrolment rate commonly observed in obesity trials.^26^ For each stratum, block size was randomly either 2 or 4. All personnel remained blinded to treatment allocation codes until database lock after 24 weeks.

### 2.3 Treatments

All participants received training in self‐administration of a subcutaneously injected placebo microsphere preparation (no active ingredient). At randomization, participants assigned to active treatment (dapagliflozin/exenatide) received dapagliflozin 10 mg once daily taken orally each morning and exenatide 2 mg once weekly extended‐release formulation administered subcutaneously on the same day each week, at any time of day (Figure 1A). Participants assigned to placebo received oral tablets matched to dapagliflozin 10‐mg tablets and self‐administered the placebo injection. First doses were given on the day of randomization after investigations were completed. Treatment compliance was assessed by quantifying the amount of investigational products returned and participant‐reported dosing.

Participants were instructed to continue regular medication (e.g. antihypertensive or lipid‐lowering agents) without changing dosages and to take no new prescription or non‐prescription medications unless advised by a physician. Participants were requested to report changes in medication or dosages to study investigators.

At each study visit, participants were given a standard leaflet and instructed to follow a balanced diet as per national guidelines. Participants were also advised to exercise moderately (e.g. walking 30 minutes most days); however, diet and exercise modification was not mandated or documented. To evaluate pharmacological effects, major lifestyle changes were not an aim of the study.

### 2.4 Outcomes

#### 2.4.1 Efficacy

The primary efficacy endpoint was change in body weight (kg) from baseline (day of randomization) to 24 weeks with dapagliflozin/exenatide versus placebo. Body weight was measured using the same electronic scale (TANITA) at baseline, 4, 8, 12 and 24 weeks, with participants in light indoor clothing without shoes. The secondary efficacy endpoint was the percentage of body weight change from baseline to 24 weeks.

Exploratory efficacy endpoints measured at baseline and 24 weeks included proportions of participants with ≥5% and ≥10% body weight reduction, body composition measures, glycaemic measures, SBP and waist/hip circumference.

At weeks 0 and 24 MRI was conducted (File S1) to assess changes in volumes of abdominal visceral adipose tissue, abdominal subcutaneous adipose tissue, total adipose tissue, total lean tissue and liver fat content.

Glycaemic measures included changes from baseline in glycated haemoglobin (HbA1c) and fasting plasma glucose (FPG), derived from blood samples taken between 07:30 and 10:30 hours after an overnight fast. A standard oral glucose tolerance test (OGTT), conducted at screening and at 24 weeks (File S1), assessed 2‐hour plasma glucose, and proportions with impaired fasting glucose (IFG; defined as an FPG ≥5.6 mmol/L, measured just before the OGTT), impaired glucose tolerance (IGT; defined as a plasma glucose value ≥7.8 mmol/L measured 120 minutes after the start of the OGTT), and prediabetes (any IFG or IGT). A dapagliflozin/placebo tablet was taken orally 30 minutes before the 24‐week OGTT.

Urine dipstick tests were performed at baseline and showed no overt glucosuria. No further urinary glucose testing was undertaken until 3‐hour urine collection during the 24‐week OGTT; 24‐week urinary glucose was analysed after study completion to maintain blinding until database lock.

Cardiovascular measures included change from baseline in seated SBP, diastolic blood pressure (DBP), which were both measured as the average of two readings and rounded to the nearest 1 mm Hg, and heart rate.

#### 2.4.2 Safety

Safety and tolerability were assessed over 24 weeks by evaluating the changes from baseline in estimated glomerular filtration rate (eGFR; estimated using the Modification of Diet in Renal Disease equation),^27^ fasting ketones, fasting serum lipids and other laboratory variables of interest. Reports of adverse events (AEs, serious AEs, study treatment‐related AEs as assessed by the investigator, AEs leading to discontinuation of study treatment) were collected and coded using the Medical Dictionary for Regulatory Activities (MedDRA) version 18.0. Furthermore, AEs of special interest for dapagliflozin and exenatide treatment (urinary tract infections, genital infections, events related to volume reduction, changes in renal function and gastrointestinal symptomatology) were captured using prespecified lists of relevant MedDRA preferred terms.

All blood and urine analyses were performed using standard assays at the Clinical Chemistry Laboratory of the Uppsala University Hospital.

### 2.5 Statistical methods

The primary, secondary and exploratory efficacy endpoints were analysed in the full analysis set (FAS), defined as participants who received ≥1 dose of study medication during the 24‐week double‐blind treatment period, who had a baseline value and ≥1 post‐baseline value for ≥1 efficacy measure during the double‐blind period, and who were not found to have serious protocol non‐compliance. The analysis of safety used the safety analysis set, defined as all randomized participants who received ≥1 dose of study medication.

For primary and secondary endpoints, least‐squares (LS) mean changes from baseline to 24 weeks and associated 95% confidence intervals (CIs) and p values for dapagliflozin/exenatide versus placebo differences were derived from a mixed model for repeated measures, with treatment, week, treatment‐by‐week interaction and sex as categorical fixed covariates, and baseline value as a continuous fixed covariate. An unstructured matrix for the within‐participant error variance‐covariance was used. A similar approach was used for changes in HbA1c, blood pressure, heart rate, eGFR and serum lipids. For MRI, FPG and fasting ketones, similar model terms were employed, but analysis of covariance was used. The significance of changes in proportions from baseline to 24 weeks for IFG and IGT was evaluated with paired McNemar tests for within‐treatment group changes and a Cochran–Mantel–Haenszel test, adjusted for treatment, for between‐treatment group changes.

For sample‐size calculations, see File S1. All statistical analyses were performed using SAS version 9.4 (SAS Institute Inc., Cary, NC, USA). CIs and p values were unadjusted for multiple comparisons.

## 3 RESULTS

### 3.1 Participants

Of 25 participants randomized to receive dapagliflozin/exenatide and 25 randomized to receive placebo, 23 (92.0%) and 20 (80.0%) completed 24 weeks of double‐blind treatment, respectively (Figure 1B). Two dapagliflozin/exenatide‐treated participants discontinued because of AEs (abdominal pain and injection‐site pruritus/mass). Three placebo‐treated participants discontinued because of AEs (skin ulcer/vasculitis, malaise and dyspnoea/fatigue). One placebo‐treated participant withdrew consent and one was excluded for protocol non‐compliance (strict low‐carbohydrate/high‐fat diet that elevated ketones, resulting in FAS exclusion). No withdrawals led to study medication unblinding.

Glycaemic variables, vital signs and renal function were balanced across treatment groups at baseline; however, mean age, body weight, duration of obesity, waist circumference and body fat measures were numerically higher at baseline in the dapagliflozin/exenatide group than in the placebo group (Table 1). Overall, more participants had IFG than IGT at baseline (Table 1).

**Table 1:** Demographic and baseline characteristics (FAS)

	Dapagliflozin 10 mg once daily + exenatide 2 mg once weekly	Placebo oral tablet once daily + placebo injection once weekly
	**n = 25**	**n = 24**
Age, years	53.5 (13.5)	50.0 (11.8)
Sex, n (%)		
Female	15 (60.0)	15 (62.5)
Male	10 (40.0)	9 (37.5)
Body weight, kg	106.43 (15.55)	102.72 (17.26)
Body mass index, kg/m^2^	35.8 (2.9)	35.0 (3.7)
Waist circumference, cm	117.6 (11.3)	114.4 (12.6)
Hip circumference, cm	121.2 (7.5)	117.6 (6.5)
Duration of obesity, years	27.3 (17.3)	19.7 (13.5)
Body composition^a^		
Visceral abdominal adipose tissue, L	6.3 (3.1)	5.7 (2.4)
Subcutaneous abdominal adipose tissue,^b^ L	14.4 (3.8)	14.0 (2.5)
Total adipose tissue, L	57.1 (9.7)	53.4 (7.0)
Total lean tissue, L	42.6 (9.6)	40.4 (9.1)
Liver fat, %	10.9 (10.6)	10.0 (8.5)
HbA1c		
mmol/mol	37.2 (3.9)	38.1 (3.3)
%	5.6 (0.35)	5.6 (0.30)
FPG, mmol/L	5.90 (0.63)	5.83 (0.43)
2‐h PG (during OGTT), mmol/L	7.99 (2.25)	7.10 (1.12)
Impaired fasting glucose,^c^ n (%)	16 (64.0)	17 (70.8)
Impaired glucose tolerance,^d^ n (%)	12 (48.0)	8 (33.3)
Vital signs		
DBP, mm Hg	75.8 (10.6)	77.0 (12.6)
SBP, mm Hg	133.7 (12.7)	133.8 (17.5)
Heart rate, bpm	67.8 (9.5)	67.0 (9.2)
eGFR,^e^ mL/min/1.73m^2^	85.0 (17.3)	84.6 (12.1)
Fasting ketones, mmol/L	0.23 (0.11)	0.21 (0.07)
Serum lipids		
Total cholesterol, mmol/L	5.34 (1.00)	5.35 (0.95)
LDL cholesterol, mmol/L	3.52 (0.91)	3.44 (0.89)
HDL cholesterol, mmol/L	1.26 (0.28)	1.26 (0.31)
Triglycerides, mmol/L	1.40 (0.57)	1.59 (0.59)
Free fatty acids, µmol/L	218 (75)	201 (43)
Comorbidities, n (%)	19 (76.0)	17 (70.8)
Gastrointestinal disorders	7 (28.0)	3 (12.5)
Musculoskeletal disorders	4 (16.0)	6 (25.0)
Respiratory and thoracic disorders	4 (16.0)	6 (25.0)
Hypertension	6 (24.0)	4 (16.7)
Depression	2 (8.0)	1 (4.2)
Other endocrine disorders^f^	5 (20.0)	3 (12.5)
Prior anti‐obesity medications,^g^ n (%)	6 (24.0)	1 (4.2)
Orlistat	4 (16.0)	1 (4.2)
Sibutramine	3 (12.0)	0 (0.0)
Concomitant medications,^h^ n (%)	24 (96.0)	23 (95.8)
ARB/ACE inhibitor	4 (16.0)	6 (25.0)
Diuretics	4 (16.0)	1 (4.2)
Thyroid replacement therapy	5 (20.0)	2 (8.3)
Calcium channel blockers	0 (0.0)	3 (12.5)
Beta‐blocking agents	0 (0.0)	1 (4.2)

Abbreviations: ACE, angiotensin converting‐enzyme; ARB, angiotensin receptor blocker; NSAID, non‐steroidal anti‐inflammatory drug; PG, plasma glucose; s.d., standard deviation.

Data are mean (s.d.), unless otherwise indicated.

a Measured using MRI.

b Defined as the subcutaneous fat positioned between the hip joint and up to the lower pole of the lungs.

c Defined as a fasting plasma glucose value ≥5.6 mmol/L measured at time = 0 of an OGTT conducted at the screening visit.

d Defined as a plasma glucose value ≥7.8 mmol/L measured at time = 120 minutes of an OGTT conducted at the screening visit.

e Assessed using the Modification of Diet in Renal Disease formula.

f Hypothyroidism (n = 6), goiter (n = 1), and partial pituitary insufficiency (n = 1).

g Stopped earlier than 1 month before enrolment.

h Ongoing or started on the date of randomization or stopped after randomization.

### 3.2 Efficacy

#### 3.2.1 Body weight

**Figure 2:**
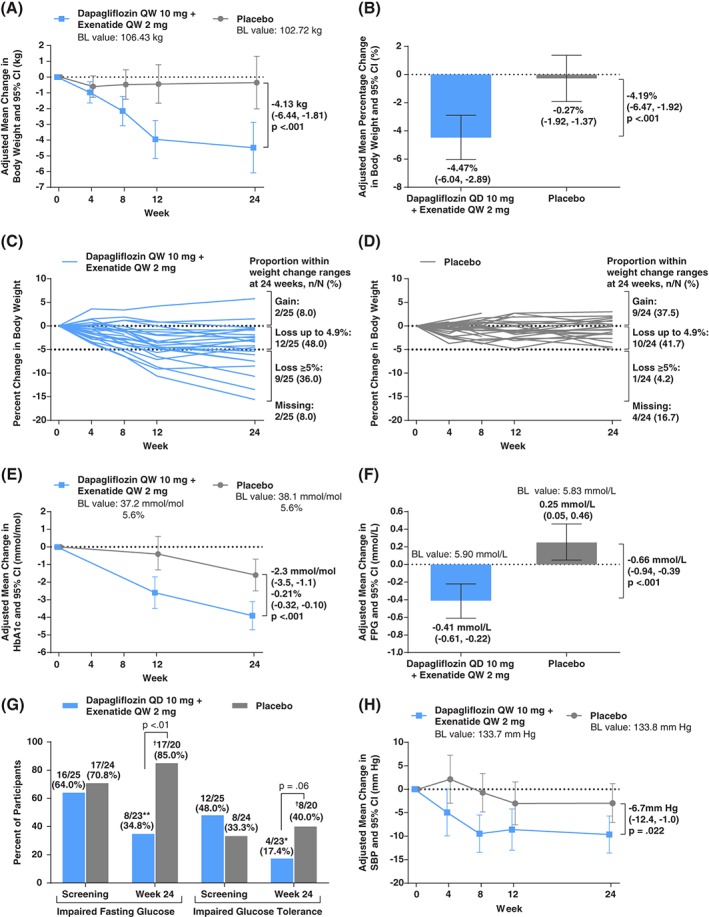
A, Primary endpoint: adjusted mean change from baseline in body weight (kg) over 24 weeks; B, secondary endpoint: adjusted mean percentage change from baseline in body weight (%) after 24 weeks; C, individual participant trajectories of percent change in body weight over 24 weeks in dapagliflozin/exenatide‐treated participants; D, corresponding trajectories in placebo‐treated participants; E, adjusted mean change from baseline in HbA1c (mmol/mol and %) over 24 weeks; F, adjusted mean change from baseline in FPG (mmol/L) after 24 weeks; G, proportions of participants with IFG or impaired glucose tolerance at screening and after 24 weeks; H, adjusted mean change from baseline in SBP (mm Hg) over 24 weeks. Analyses in panels A, B, E, and H used mixed models for repeated measures of change or percentage change from baseline adjusted for treatment, week, treatment‐by‐week, sex, and baseline value. The analysis in panel F used analysis of covariance of change from baseline adjusted for treatment, week, treatment‐by‐week, sex and baseline value. Treatment differences presented in panels A, B, E, F, and H are adjusted LS mean differences with 95% CIs. ^*^ *P* < .05; ^**^ *P* < .01 (compared to baseline). The analyses in all panels used the FAS; in panels C and D, corresponding per‐protocol analysis set proportions within weight loss ranges at 24 weeks (weight gain, weight loss up to 4.9%, weight loss ≥5%) were 2/22 (9.1%), 11/22 (50.0%), and 9/22 (40.9%) with dapagliflozin/exenatide, and 9/20 (45.0%), 10/20 (50.0%), and 1/20 (5.0%) with placebo. ^†^One of these placebo‐treated participants was diagnosed with type 2 diabetes based on American Diabetes Association plasma glucose and HbA1c criteria at the final 24‐week visit. BL, baseline.

Dapagliflozin/exenatide reduced body weight significantly more than placebo (Figure 2A; Table 2), with a mean difference of −4.13 kg after 24 weeks. The overall percentage reduction in body weight was also significantly greater with dapagliflozin/exenatide than placebo (Figure 2B; Table 2), and more participants treated with dapagliflozin/exenatide than placebo achieved body weight reductions ≥5% and ≥10% of their initial weight (Table 2). Similarly, more participants treated with dapagliflozin/exenatide than placebo achieved any body weight loss or no body weight gain (Figure 2C,D). The rate of body weight loss with dapagliflozin/exenatide appeared to be most rapid up to 12 weeks for most participants, although some showed continuing body weight loss to 24 weeks (Figure 2C).

**Table 2:** Primary, secondary and exploratory endpoints after 24 weeks (FAS)

	**Dapagliflozin 10 mg once daily + exenatide 2 mg once weekly**	**Placebo oral tablet once daily + placebo injection once weekly**	**Dapagliflozin/exenatide vs placebo difference**	***P* value**
	**(n = 25)**	**(n = 24)**		
Primary endpoint^a^				
Body weight, adjusted mean change (95% CI), kg	−4.48 (−6.08, −2.87)	−0.35 (−2.02, 1.32)	−4.13 (−6.44, −1.81)	<.001
Secondary endpoint^a^				
Body weight, adjusted mean percentage change (95% CI), %	−4.47 (−6.04, −2.89)	−0.27 (−1.92, 1.37)	−4.19 (−6.47, −1.92)	<.001
Exploratory endpoints				
Body weight, proportion with ≥5% reduction, n (%)	9 (36.0)	1 (4.2)		
Body weight, proportion with ≥10% reduction, n (%)	3 (12.0)	0 (0.0)		
MRI body composition measures^b^				
Visceral abdominal adipose tissue, adjusted mean change (95% CI), l	−0.40 (−0.63, −0.16)	0.22 (−0.02, 0.46)	−0.62 (−0.95, −0.29)	<.001
Subcutaneous abdominal adipose tissue,^c^ adjusted mean change (95% CI), l	−1.31 (−1.80, −0.81)	0.13 (−0.41, 0.67)	−1.44 (−2.16, −0.71)	<.001
Total adipose tissue, adjusted mean change (95% CI), l	−4.22 (−5.69, −2.76)	−0.14 (−1.72, 1.45)	−4.09 (−6.23, −1.94)	<.001
Total lean tissue, adjusted mean change (95% CI), l	−0.91 (−1.45, −0.37)	−0.72 (−1.30, −0.14)	−0.19 (−0.94, 0.56)	.614
Liver fat, adjusted mean percentage change (95% CI), %	−1.41 (−2.75, −0.08)	−0.31 (−1.71, 1.08)	−1.10 (−3.01, 0.82)	.254
Glycaemic measures				
HbA1c, adjusted mean change (95% CI),^a^ mmol/mol	−3.9 (−4.7, −3.1)	−1.6 (−2.5, −0.7)	−2.3 (−3.5, −1.1)	<.001
HbA1c, adjusted mean change (95% CI),^a^ %	−0.36 (−0.43, −0.28)	−0.15 (−0.23, −0.06)	−0.21 (−0.32, −0.10)	<.001
FPG, adjusted mean change (95% CI),^b^ mmol/l	−0.41 (−0.61, −0.22)	0.25 (0.05, 0.46)	−0.66 (−0.94, −0.39)	<.001
2‐h PG, adjusted mean change (95% CI),^b^ mmol/L	−1.57 (−2.36, −0.78)	−0.08 (−0.93, 0.76)	−1.49 (−2.65, −0.33)	.013
Proportion with IFG, n/N (%)	8/23 (34.8)	17/20 (85.0)		<.001^d^
Proportion with IGT, n/N (%)	4/23 (17.4)	8/20 (40.0)		.066^d^
Proportion with any IFG or IGT (prediabetes), n/N (%)	8/23 (34.8)	17/20 (85.0)		.002^d^
Vital signs^a^				
DBP, adjusted mean change (95% CI), mm Hg	1.8 (−2.7, 6.3)	2.3 (−2.6, 7.1)	−0.5 (−7.1, 6.1)	.883
SBP, adjusted mean change (95% CI), mm Hg	−9.6 (−13.6, −5.7)	−3.0 (−7.1, 1.2)	−6.7 (−12.4, −1.0)	.022
Heart rate, adjusted mean change (95% CI), bpm	2.6 (−0.1, 5.3)	0.4 (−2.5, 3.3)	2.1 (−1.8, 6.1)	.281
Waist circumference, adjusted mean change (95% CI),^a^ cm	−5.3 (−7.5, −3.1)	−2.6 (−4.8, −0.3)	−2.8 (−5.9, 0.4)	.083
Waist‐to‐hip ratio, adjusted mean change (95% CI)^a^	−0.02 (−0.04, 0.000)	−0.01 (−0.03, 0.01)	−0.01 (−0.03, 0.02)	.710
Urinary glucose excretion^e^				
Mean (s.d.), mmol/3 h	50.5 (31.4)	0.26 (0.8)		

Abbreviation: PG, plasma glucose.

IFG defined as an FPG ≥5.6 mmol/L measured just before an OGTT at the 24‐week visit). IGT defined as a plasma glucose value ≥7.8 mmol/L measured 2 hours after the start of an OGTT at the 24‐week visit; 2‐hour PG, measured 2 hours after the start of the OGTT.

a Data are LS mean changes from baseline to 24 weeks and 95% CIs derived from a mixed model for repeated measures adjusted for treatment, week, treatment‐by‐week, sex, and baseline value.

b Data are LS mean changes from baseline to 24 weeks and 95% CIs derived from an analysis of covariance model adjusted for treatment, sex, and baseline value.

c Defined as the subcutaneous fat positioned between the hip joint and up to the lower pole of the lungs.

d p value based on a Cochran–Mantel–Haenszel test adjusted for treatment.

e Values at 24 weeks derived from urine collected during a 3‐hour OGTT.

#### 3.2.2 MRI of body composition

Dapagliflozin/exenatide reduced visceral, subcutaneous and total adipose tissue volume, but not lean tissue, significantly more than placebo. Total adipose tissue reduction versus placebo was −4.09 L after 24 weeks (Table 2). Although dapagliflozin/exenatide significantly reduced percent liver fat from baseline, this reduction was not significantly different from placebo (Table 2).

#### 3.2.3 Glycaemic variables

Dapagliflozin/exenatide reduced HbA1c, FPG and 2‐hour plasma glucose significantly more than placebo over 24 weeks (Table 2; Figure 2E,F), with mean differences of −2.3 mmol/mol (−0.21%), −0.66 and −1.49 mmol/L, respectively.

Although the participants did not have diabetes, OGTTs showed that many had abnormal glucose metabolism at baseline, which improved with dapagliflozin/exenatide but not placebo. The proportion of dapagliflozin/exenatide‐treated participants with IFG was significantly reduced, from 64.0% at baseline to 34.8% at week 24 (p = .008), and with IGT was significantly reduced from 48.0% to 17.4% (p = .020). Combined, the proportion of participants with prediabetes (any IFG or IGT) significantly decreased from 68.0% to 34.8% (p = .005). In contrast, the corresponding proportions among placebo‐treated participants did not change significantly from baseline to 24 weeks (70.8%–85.0%, 33.3%–40.0% and 79.2%–85.0%, respectively; all *P* > .3; Figure 2G). Reduction in prediabetes (any IFG or IGT) from baseline to 24 weeks was significantly greater with dapagliflozin/exenatide than placebo (p = .002).

No participants had overt glucosuria at baseline. After 24 weeks, mean urinary glucose was substantially greater with dapagliflozin/exenatide (50.5 mmol/3 h) than with placebo (0.3 mmol/3 h).

#### 3.2.4 Vital signs

In this predominantly normotensive population (mean baseline SBP of 134 mm Hg), dapagliflozin/exenatide reduced SBP significantly more than placebo (Figure 2H), with a mean difference of −6.7 mm Hg after 24 weeks. No significant differences between dapagliflozin/exenatide and placebo were observed for DBP or heart rate (Table 2). Some participants, mainly in the dapagliflozin/exenatide group, had pronounced reductions in SBP; however, this did not lead to withdrawal or dose reduction of antihypertensive drugs among participants treated for hypertension. Hypotension was reported in only one placebo‐treated participant. New antihypertensive therapies were started in three participants, one in the dapagliflozin/exenatide group and two in the placebo group.

### 3.3 Safety and tolerability

No meaningful differences between dapagliflozin/exenatide and placebo were observed for eGFR, fasting ketones or serum lipids (Table 3), or other safety laboratory variables (Table S1).

**Table 3:** Key laboratory changes and safety after 24 weeks

	**Dapagliflozin 10 mg once daily + exenatide 2 mg once weekly**	**Placebo oral tablet once daily + placebo injection once weekly**	**Dapagliflozin/exenatide vs placebo difference**	***P* value**
Laboratory variables analysed using the FAS	n = 25	n = 24		
eGFR,^a^ adjusted mean change (95% CI),^b^ mL/min/1.73 m^2^	−1.97 (−6.52, 2.59)	2.47 (−2.65, 7.59)	−4.44 (−11.22, 2.35)	.193
Fasting ketones, adjusted mean change (95% CI),^c^ mmol/L	0.01 (−0.03, 0.06)	−0.03 (−0.08, 0.01)	0.05 (−0.02, 0.11)	.149
Serum lipids, adjusted mean change (95% CI)				
Total cholesterol,^b^ mmol/L	−0.17 (−0.42, 0.09)	−0.26 (−0.54, 0.01)	0.10 (−0.27, 0.47)	.596
LDL cholesterol,^b^ mmol/L	−0.17 (−0.38, 0.04)	−0.22 (−0.44, −0.00)	0.05 (−0.25, 0.35)	.727
HDL cholesterol,^b^ mmol/L	0.02 (−0.06, 0.09)	−0.07 (−0.15, 0.01)	0.09 (−0.02, 0.20)	.101
Triglycerides^b^ mmol/L	−0.10 (−0.29, 0.10)	−0.01 (−0.21, 0.20)	−0.09 (−0.37, 0.19)	.516
Free fatty acids,^c^ µmol/L	−0.8 (−19.9, 18.3)	−13.5 (−34.2, 7.1)	12.8 (−15.2, 40.7)	.361
Safety variables analysed using the safety analysis set	n = 25	n = 25		
Participants with at least one AE, n (%)				
Any AE	25 (100.0)	25 (100.0)		
Any serious AEs	1 (4.0)	1 (4.0)		
Treatment‐related AEs	4 (16.0)	3 (12.0)		
AEs leading to study discontinuation	2 (8.0)	3 (12.0)		
Deaths	0 (0.0)	0 (0.0)		
AEs of special interest^d^				
Urinary tract infections	2 (8.0)	1 (4.0)		
Acute pyelonephritis	1 (4.0)	0 (0.0)		
Urinary tract infection	0 (0.0)	1 (4.0)		
Fungal urinary tract infection	1 (4.0)	0 (0.0)		
Genital infections	1 (4.0)	0 (0.0)		
Vaginal infection	1 (4.0)	0 (0.0)		
Volume reduction	0 (0.0)	1 (4.0)		
Hypotension	0 (0.0)	1 (4.0)		
Renal impairment/failure	0 (0.0)	0 (0.0)		
Gastrointestinal symptoms	16 (64.0)	10 (40.0)		
Nausea	7 (28.0)	3 (12.0)		
Diarrhea	3 (12.0)	3 (12.0)		
Abdominal distension	3 (12.0)	2 (8.0)		
Vomiting	3 (12.0)	1 (4.0)		
Gastroesophageal reflux	3 (12.0)	1 (4.0)		
Constipation	2 (8.0)	1 (4.0)		
Dyspepsia	2 (8.0)	0 (0.0)		
Abdominal pain	1 (4.0)	0 (0.0)		
Injection‐site disorders	11 (44.0)	8 (32.0)		
Injection‐site mass	7 (28.0)	5 (20.0)		
Injection‐site pruritus	7 (28.0)	2 (8.0)		
Injection‐site erythema	3 (12.0)	1 (4.0)		
Injection‐site nodule	2 (8.0)	1 (4.0)		
Injection‐site swelling	0 (0.0)	2 (8.0)		
Injection‐site pain	1 (4.0)	0 (0.0)		
Injection‐site cyst	1 (4.0)	0 (0.0)		
Injection‐site rash	0 (0.0)	1 (4.0)		
Appetite changes	10 (40.0)	6 (24.0)		
Decreased appetite	8 (32.0)	3 (12.0)		
Increased appetite	1 (4.0)	0 (0.0)		
Hunger	1 (4.0)	3 (12.0)		

a Assessed using the Modification of Diet in Renal Disease formula.

b Data are LS mean changes from baseline to 24 weeks and 95% CIs derived from a mixed model for repeated measures adjusted for treatment, week, treatment‐by‐week, sex, and baseline value.

c Data are LS mean changes from baseline to 24 weeks and 95% CIs derived from an analysis of covariance model adjusted for treatment, sex, and baseline value.

d AEs of special interest for dapagliflozin and exenatide (e.g. urinary tract and genital infections and vomiting, nausea, and diarrhoea, respectively) were coded using predefined lists of MedDRA version 18.0 preferred terms.

All participants reported at least one AE (Table 3). Serious AEs occurred in two participants [hospitalization from head trauma (dapagliflozin/exenatide) and hospitalization because of dyspnoea (placebo)]. No deaths occurred. The most common AEs occurring in dapagliflozin/exenatide‐ and placebo‐treated participants, respectively, were nasopharyngitis, nine (36.0%) and four (16.0%), headache, eight (32.0%) and four (16.0%), and decreased appetite, eight (32.0%) and three (12.0%). No participant experienced confirmed hypoglycaemia. One placebo‐treated participant was diagnosed with T2D at the final 24‐week visit.

Urinary tract infections or genital infections were reported in three participants receiving dapagliflozin/exenatide and one participant receiving placebo; no participants reported AEs potentially related to renal impairment/failure (Table 3).

Gastrointestinal and injection‐site‐related AEs were more common with dapagliflozin/exenatide than placebo. Gastrointestinal AEs occurred in 16 (64.0%) and 10 (40.0%) of dapagliflozin/exenatide‐ and placebo‐treated participants, respectively; nausea was the most frequent, occurring in seven (28.0%) and three (12.0%) participants, respectively. Injection‐site‐related AEs occurred in 11 (44.0%) and eight (32.0%) of dapagliflozin/exenatide‐ and placebo‐treated participants, respectively (Table 3).

## 4 DISCUSSION

Classes of glucose‐lowering agents for T2D treatment that reduce body weight include SGLT2 inhibitors and GLP‐1RAs. These two drug classes have rarely been evaluated together in T2D trials and their dual effects on body weight loss have not been previously studied in obese adults without diabetes. The results of the present study showed that dual therapy with dapagliflozin 10 mg once daily and exenatide 2 mg once weekly significantly reduced body weight in obese adults without diabetes over 24 weeks of follow‐up, with most changes observed during the first 12 weeks. Among dapagliflozin/exenatide‐treated participants, 36.0% lost ≥5% and 12.0% lost ≥10% of their initial weight. MRI showed that body weight loss was primarily adipose rather than lean tissue, with an average total adipose tissue loss of 4.09 L. HbA1c, FPG and the proportion of participants with prediabetes were also improved with dapagliflozin/exenatide versus placebo. Dapagliflozin/exenatide reduced SBP by −6.7 mm Hg versus placebo over 24 weeks. Dapagliflozin/exenatide was well tolerated with a side‐effect profile consistent with that observed in trials of the individual agents. No unexpected safety concerns were identified.

Body weight loss observed after dual therapy appeared to be greater than for dapagliflozin and exenatide individually in previous T2D studies,^12^, ^21^ and these drugs may have contributed additively to the observed body weight loss. Dapagliflozin reduces body weight via increased calorie expenditure through the urine; however, this calorie loss can lead to a compensatory appetite increase.^8^ In contrast, exenatide reduces appetite and delays gastric emptying, which both lead to bodyweight loss.^28^ Used together, the mechanisms of the two drugs may have complemented each other; however, the potential for an additive effect cannot be evaluated because this trial did not contain monotherapy comparator arms.

The combination of a selective SGLT2 inhibitor and a GLP‐1RA initiated together has not been studied previously, but available data on sequential addition of these drug classes in patients with T2D support this treatment approach. In the randomized, double‐blind, placebo‐controlled CANVAS study, patients failing treatment for diabetes with an incretin mimetic (dipeptidyl peptidase‐4 inhibitor or GLP‐1RA) received the SGLT2 inhibitor canagliflozin or placebo.^29^ After 18 weeks, placebo‐subtracted reductions in body weight were −2.7 and −3.3 kg with canagliflozin 100 and 300 mg, respectively, both added to a GLP‐1RA. HbA1c and SBP were also reduced for both doses of canagliflozin added to a GLP‐1RA.

Ongoing clinical trials investigating the SGLT2 inhibitor/GLP‐1RA combination initiated together in patients with T2D will provide important information on body weight loss in T2D. These include DURATION‐8 (NCT02229396), evaluating exenatide 2 mg once weekly and dapagliflozin 10 mg once daily, administered as single agents and as dual therapy in patients withT2D with inadequate glycaemic control on metformin, and AWARD‐10 (NCT02597049), evaluating empagliflozin added sequentially to dulaglutide.

Identification of the type of tissue lost during treatment with dapagliflozin/exenatide is important because lean tissue loss would be less beneficial to obese adults than adipose tissue loss, in particular visceral or ectopic fat deposits. In the present study, compared with placebo, dapagliflozin/exenatide treatment significantly reduced subcutaneous and visceral abdominal adipose tissue (but not lean tissue). An earlier study indicated that the body weight lost with dapagliflozin 10 mg once daily added to metformin in patients with T2D was two‐thirds fat mass and one‐third lean mass.^14^ A study in which immediate‐release exenatide was added to metformin found that body weight loss over 1 year with this combination reduced total fat mass by −11% (approximately −2.4 kg) among 29 patients with T2D without affecting lean mass.^22^ Exenatide treatment has also been reported to reduce liver fat over 6 months by −18%.^30^ The adipose tissue reductions found in this study are generally compatible with previous observations of dapagliflozin and exenatide individually, although the numeric reduction in liver fat with dapagliflozin/exenatide was not significantly different from placebo. Because the present study was not powered to detect differences in liver fat, this merits further exploration, e.g. in populations with more pronounced hepatic steatosis at baseline.

Hypertension is a frequent comorbidity of obesity and diabetes and an important part of metabolic syndrome. Improving blood pressure control is a common clinical goal for obese patients. Individually, dapagliflozin 10 mg once daily has been associated with SBP reductions ranging from −2.6 to −3.6 mm Hg^31^ and exenatide (pooled data of immediate‐ and extended‐release preparations) with SBP reductions of −1.8 mm Hg.^32^ Importantly, the observed reduction in SBP of −6.7 mm Hg in the present study was larger than would have been expected from either drug component administered individually.^31^, ^32^ Dapagliflozin can reduce SBP via several mechanisms, including reduction in body weight^9^ and increased osmotic diuresis and natriuresis.^10^ The mechanism by which exenatide reduces SBP is less well understood but may involve vasodilation^33^ and/or natriuresis.^34^ Thus, the SBP‐lowering mechanisms of exenatide may complement or augment dapagliflozin‐induced plasma volume reduction. Notably, no hypotension or dehydration was reported in dapagliflozin/exenatide‐treated participants.

This study was too small to identify uncommon AEs. Nevertheless, no new safety findings, and no marked increase in discontinuations or expected AEs, were observed with SGLT2 inhibitor/GLP‐1RA dual therapy. Urinary tract infections (n = 2; 8.0%), genital infections (n = 1; 4.0%), and renal AEs (0%) were infrequent with dapagliflozin/exenatide but, as anticipated, nausea, injection‐site mass, and injection‐site pruritus appeared more frequent with dapagliflozin/exenatide (28.0%, 28.0% and 28.0%, respectively) than with placebo (12.0%, 20.0% and 8.0%, respectively).

Use of a high‐dose GLP‐1RA has been suggested as an alternative to combination pharmacotherapy for body weight loss, but the incidence of AEs and need for additional injections may limit its use. Liraglutide has been approved as a body weight loss therapy at a higher dose (3 mg once daily) than used to treat T2D (1.8 mg once daily). In a placebo‐controlled study in obese participants without diabetes receiving liraglutide 3 mg once daily, body weight loss and relative reductions in prediabetes prevalence with liraglutide appeared to be similar to those observed in the present study, but nausea occurred more frequently.^35^ Interestingly, dapagliflozin/exenatide dual therapy at higher doses may provide additional body weight loss and metabolic benefits.

The present study has some limitations. It was a small, short‐term, single‐centre, proof‐of‐concept study with no monotherapy comparator groups, which are needed to formally evaluate whether effects with dapagliflozin/exenatide on body weight loss, glycaemic control and SBP are additive. Body weight responses to dapagliflozin/exenatide varied between individuals and further research is required to identify behaviours or biomarkers predicting response to dapagliflozin/exenatide. Moreover, different results might be observed in patients undergoing intensive lifestyle intervention. The study was conducted in participants without diabetes, and results cannot be directly extrapolated to patients with T2D.

In summary, this proof‐of‐concept study shows that dapagliflozin/exenatide reduces body weight, prediabetes occurrence and SBP compared with placebo and is well tolerated in an obese population without diabetes. These findings, together with the convenience of once‐daily oral dosing and once‐weekly injection, support further investigation to assess dapagliflozin/exenatide for body weight loss and prevention of diabetes in obese individuals.

## Supporting information


**File S1.** Inclusion/Exclusion Criteria; MRI methods; OGTT methods; sample size calculations; Table S1 (other laboratory parameters).
