# Supplementary material for: Dapagliflozin once‐daily and exenatide once‐weekly dual therapy: A 24‐week randomized, placebo‐controlled, phase II study examining effects on body weight and prediabetes in obese adults without diabetes
Source: Diabetes Obes Metab. 2016 Sep 26;19(1):49–60. doi: 10.1111/dom.12779 (PMC5215525; doi:10.1111/dom.12779)
Supplement: Supplementary file 1 — File S1. Inclusion/Exclusion Criteria; MRI methods; OGTT methods; sample size calculations; Table S1 (other laboratory parameters). [file DOM-19-49-s001.docx]

# Supporting Information

## Inclusion and Exclusion Criteria

For inclusion in the study, participants should fulfill the following criteria:

1. Provision of signed informed consent prior to any study-specific procedures
2. Female and/or male aged 18 to 70 years with body mass index (BMI; measured as bodyweight (kg)/(height (m))^2^) 30 to 45 kg/m^2^
3. Female participants must meet all of the following criteria:
   1. Not breastfeeding
   2. Negative pregnancy test result (human chorionic gonadotropin, beta subunit) at Visit 1 (Enrollment) (not applicable to hysterectomized females)
   3. If of childbearing potential (including perimenopausal women who have had a menstrual period within 1 year), must practice and be willing to continue to practice one of the following highly effective birth control methods during the entire duration of the study:
      1. Diaphragm or partner use of condom in combination with combined (estrogen- and progestogen-containing) hormonal contraception associated with inhibition of ovulation:
         - Oral
         - Intravaginal
         - Transdermal
      2. Diaphragm or partner use of condom in combination with progestogen-only hormonal contraception associated with inhibition of ovulation:
         - Oral
         - Injectable
         - Implantable
      3. Placement of an intrauterine device
      4. Placement of an intrauterine hormone-releasing system
      5. Bilateral tubal occlusion
      6. Vasectomized partner (provided that the partner is the sole sexual partner of the female participant and that the vasectomized partner has received medical assessment of the surgical success)
      7. Sexual abstinence (defined as refraining from heterosexual intercourse)
   4. Must practice appropriate birth control as stated above for 10 weeks after the last dose of study medication

Participants should not enter the study if any of the following exclusion criteria are fulfilled:

1. Involvement in the planning and/or conduct of the study
2. Previous enrollment in the present study
3. Participation in another clinical study with an investigational product during the last 3 months prior to Visit 1
4. History of any clinically significant disease, disorder, or condition which, in the opinion of the Investigator, may either put the participant at risk because of participation in the study or influence the results or the participant’s ability to participate in the study
5. Previous or new diagnosis of diabetes mellitus. For participants being diagnosed with diabetes at enrollment, this should be judged by an experienced diabetologist and be based on composite laboratory measures according to American Diabetes Association guidelines. These criteria include fasting plasma glucose (FPG) >7.0 mmol/L, 2-hour postprandial glucose (PPG) at oral glucose tolerance test >11.1 mmol/L and/or glycated hemoglobin (HbA1c) >48 mmol/mol. Participants with FPG ≥7.0 mmol/L or 2-hour PPG ≥11.1 mmol/L at Visit 1, should have a second FPG measurement on a separate day; if diabetes diagnosis is confirmed, the participant will be excluded
6. Any clinically significant abnormalities in physical examination or clinical chemistry results as judged by the Investigator. The following specific exclusion criteria apply to the selected Clinical Chemistry results:
   1. Creatinine clearance <60 mL/min (estimated with Cockcroft-Gault formula)
   2. Severe hepatic insufficiency and/or significant abnormal liver function defined as aspartate aminotransferase >3 × upper limit of normal (ULN) and/or alanine aminotransferase >3 × ULN
   3. Total bilirubin >2.0 mg/dL (34.2 μmol/L)
7. Positive serologic evidence of current infectious liver disease including hepatitis B viral antibody IgM, hepatitis B surface antigen, and hepatitis C virus antibody
8. Volume-depleted individuals. Individuals at risk for volume depletion due to co-existing conditions or concomitant medications, such as loop diuretics, should have careful monitoring of their volume status
9. Acute coronary syndrome within 2 months prior to Visit 1. Hospitalization for unstable angina or acute myocardial infarction within 2 months prior to enrollment. Acute stroke or transient ischemic attack within 2 months prior to Visit 1. Less than 2 months post coronary artery revascularization
10. History of gastroparesis or pancreatitis
11. History of malignancy within the last 5 years, excluding successful treatment of basal or squamous cell skin cancer
12. Bodyweight loss greater than 5% within 3 months prior to Visit 1
13. Treatment with any drug known to affect bodyweight within the last month (e.g. systemic glucocorticoids, antipsychotics, or orlistat)
14. Multiple endocrine neoplasia syndrome type 2
15. Personal or family history of medullary thyroid carcinoma

## Magnetic Resonance Imaging Methods

Magnetic resonance imaging (MRI) was used to quantify body composition in terms of adipose tissue volumes of the total (TAT), visceral (VAT), and abdominal subcutaneous (SAT; defined as the subcutaneous fat positioned between the hip joint and the lower pole of the lungs) depots as well as the total lean tissue (TLT) volume, liver fat content, and liver volume. The imaging was performed using a 1.5T Achieva clinical scanner (Philips Healthcare, Best, The Netherlands). TAT, VAT, SAT, and TLT were quantified using automated segmentation of a whole-body water-fat separated MRI scan using a previously described methodology (Kullberg 2009). Despite using a standardized positioning scheme, the limited field of view 530 × 377 × 2000 mm (sagittal × coronal × axial) sometimes cropped the feet or hands differently during the longitudinal examinations. This was corrected for by manual segmentation and exclusion of the anatomy so the same body volume was studied during the repeated visits. The quantification of liver fat was performed using manual segmentation of the entire liver from a single breath-hold water-fat scan covering a large and fixed volume of the liver. Liver volume was assessed using a dedicated scan with large volume coverage in combination with semi-automated segmentation. Collection and analyses of the MRI data were performed at one center under blinded conditions.

**Reference**

Kullberg J, Johansson L, Ahlstrom H et al. Automated assessment of whole-body adipose tissue depots from continuously moving bed MRI: a feasibility study. J Magn Reson Imaging 2009; 30: 185–193.

## Oral Glucose Tolerance Test

The procedure for conducting the oral glucose tolerance test (OGTT) at screening and at week 24 was as follows. The test was started between 7:30 and 10:30 AM. Fasting blood samples were taken just prior to ingestion of glucose solution, and they were analyzed for: hematology, clinical chemistry, glucose, HbA1c, insulin, glucagon, glycerol, ketones, C-peptide, free fatty acids, total cholesterol, triglycerides, high-density lipoprotein cholesterol, low-density lipoprotein cholesterol, and exploratory biomarker analysis.

At Time 0 minutes, when all samples were taken, the oral glucose solution (75 grams of glucose in 300-mL water) was administered and was to be consumed within 5 minutes.

Thereafter, blood samples were taken at the following time points: 15, 30, 60, 90, 120, and 180 minutes after ingestion of the oral glucose solution. At each time point, blood samples were taken for glucose and for exploratory hormonal and metabolic measurements.

The urine produced during OGTT was to be collected. Therefore, participants were instructed to void just before the glucose ingestion (Time 0) and this urine was discarded. Then, the participant was asked to void again at 180 minutes in a container. The total volume of urine collected during the 3-hour OGTT period was measured and recorded. Ten mL of urine was taken from the container and frozen for glucose measurement and calculation of glucose excretion at the Clinical Chemistry Laboratory. At baseline, no participants had glucosuria assessed with urine dipstick, and urinary glucose was not quantified during the OGTT. At 24 weeks, however, urine was collected during the OGTT for glucose measurements, as described above.

## Sample Size Calculations

Sample size calculations were based on previous studies with dapagliflozin and exenatide suggesting that their additive effect would result in approximately 4 kg of bodyweight reduction versus placebo after 24 weeks of treatment. Because no data on bodyweight reduction with dapagliflozin in nondiabetic participants was available when this trial was designed, a conservative level of 4 kg for the standard deviation of change in bodyweight was selected for the sample size calculation. To detect a difference of 4 kg between the treatment groups, 17 evaluable participants per treatment group were required for 80% power at a two-sided significance level of 0.05. Allowing for 10% data loss due to missing data (e.g. lost to follow-up), and a potentially lower treatment effect of dapagliflozin in nondiabetic participants, 24 participants per treatment arm were required.

## Supplemental Table S1. Other laboratory parameters (Safety Analysis Set).

|  | **Dapagliflozin 10 mg QD + exenatide 2 mg QW (n=25)** | **Placebo oral tablet QD + placebo injection QW (n=25)** |
| --- | --- | --- |
| **Hemoglobin, g/L** |  |  |
| Mean (SD) at screening | 140.2 (15.9) | 143.9 (12.5) |
| Mean (SD) change from screening to 24 weeks | 3.4 (12.3) | −1.8 (7.6) |
| **Creatinine, μmol/L** |  |  |
| Mean (SD) at screening | 73.3 (14.5) | 73.0 (11.6) |
| Mean (SD) change from screening to 24 weeks | 1.6 (7.6) | −1.2 (6.0) |
| **AST, µkat/L** |  |  |
| Mean (SD) at screening | 0.54 (0.19) | 0.53 (0.15) |
| Mean (SD) change from screening to 24 weeks | −0.06 (0.14) | −0.09 (0.13) |
| **ALT, µkat/L** |  |  |
| Mean (SD) at screening | 0.63 (0.38) | 0.67 (0.43) |
| Mean (SD) change from screening to 24 weeks | −0.05 (0.24) | −0.13 (0.38) |
| **Albumin, g/L** |  |  |
| Mean (SD) at screening | 37.6 (2.2) | 38.4 (2.5) |
| Mean (SD) change from screening to 24 weeks | 0.2 (2.0) | −0.1 (1.7) |
| **Sodium, mmol/L** |  |  |
| Mean (SD) at screening | 142.0 (1.6) | 142.7 (1.3) |
| Mean (SD) change from screening to 24 weeks | −0.23 (1.34) | −0.62 (1.56) |
| **Potassium, mmol/L** |  |  |
| Mean (SD) at screening | 3.8 (0.3) | 3.7 (0.3) |
| **Potassium, mmol/L** |  |  |
| Mean (SD) at screening | 3.8 (0.3) | 3.7 (0.3) |
| Mean (SD) change from screening to 24 weeks | −0.03 (0.21) | 0.12 (0.16) |
| **High-sensitivity C-reactive protein, mg/L** |  |  |
| Mean (SD) at screening | 3.36 (2.51) | 3.37 (2.65) |
| Mean (SD) change from screening to 24 weeks | 0.09 (2.17) | −0.81 (2.25) |

ALT, alanine aminotransferase; AST, aspartate aminotransferase; QD, once daily; QW, once weekly; SD, standard deviation.
